# Assessing polar bear (*Ursus maritimus*) population structure in the Hudson Bay region using SNPs

**DOI:** 10.1002/ece3.2563

**Published:** 2016-10-28

**Authors:** Michelle Viengkone, Andrew Edward Derocher, Evan Shaun Richardson, René Michael Malenfant, Joshua Moses Miller, Martyn E. Obbard, Markus G. Dyck, Nick J. Lunn, Vicki Sahanatien, Corey S. Davis

**Affiliations:** ^1^ Department of Biological SciencesUniversity of AlbertaEdmontonABCanada; ^2^ Wildlife Research DivisionScience and Technology BranchEnvironment and Climate Change CanadaUniversity of AlbertaEdmontonABCanada; ^3^ Department of BiologyUniversity of New BrunswickFrederictonNBCanada; ^4^ Department of Ecology and Evolutionary BiologyYale UniversityNew HavenCTUSA; ^5^ Wildlife Research and Monitoring SectionOntario Ministry of Natural Resources and ForestryTrent UniversityPeterboroughONCanada; ^6^ Department of EnvironmentGovernment of NunavutIgloolikNUCanada

**Keywords:** management, polar bear, population structure, single‐nucleotide polymorphisms, *Ursus maritimus*

## Abstract

Defining subpopulations using genetics has traditionally used data from microsatellite markers to investigate population structure; however, single‐nucleotide polymorphisms (SNPs) have emerged as a tool for detection of fine‐scale structure. In Hudson Bay, Canada, three polar bear (*Ursus maritimus*) subpopulations (Foxe Basin (FB), Southern Hudson Bay (SH), and Western Hudson Bay (WH)) have been delineated based on mark–recapture studies, radiotelemetry and satellite telemetry, return of marked animals in the subsistence harvest, and population genetics using microsatellites. We used SNPs to detect fine‐scale population structure in polar bears from the Hudson Bay region and compared our results to the current designations using 414 individuals genotyped at 2,603 SNPs. Analyses based on discriminant analysis of principal components (DAPC) and STRUCTURE support the presence of four genetic clusters: (i) Western—including individuals sampled in WH, SH (excluding Akimiski Island in James Bay), and southern FB (south of Southampton Island); (ii) Northern—individuals sampled in northern FB (Baffin Island) and Davis Strait (DS) (Labrador coast); (iii) Southeast—individuals from SH (Akimiski Island in James Bay); and (iv) Northeast—individuals from DS (Baffin Island). Population structure differed from microsatellite studies and current management designations demonstrating the value of using SNPs for fine‐scale population delineation in polar bears.

## 1 Introduction

Genetic techniques have been adopted by evolutionary, ecological, and conservation biologists to delineate subpopulations (Allendorf, Hohenlohe, & Luikart, 2010; Morin, Luikart, & Wayne, 2004). Until recently, most delineation studies used genetic information from microsatellite markers. The use of microsatellites to examine genetic structure is based on their high levels of polymorphism and information content per locus (Balloux & Lugon‐Moulin, 2002; Seddon, Parker, Ostrander, & Ellegren, 2005; Vignal, Milan, SanCristobal, & Eggen, 2002). However, with increasing interest in the use of larger numbers of markers, biallelic single‐nucleotide polymorphisms (SNPs) are an emerging tool in part because genotyping is almost fully automated with low error rates (Brumfield, Beerli, Nickerson, & Edwards, 2003; Morin et al., 2004; Vignal et al., 2002).

Thus far, SNPs have been useful in identifying individuals and pedigree relationships, assessing genetic diversity, and detecting gene flow (Seddon et al., 2005). However, the use of SNPs for most wildlife species has been limited due to the high costs associated with discovery and typing of large marker sets (Vignal et al., 2002). However, the onset of new techniques to discover and genotype large marker sets simultaneously with methods like genotyping‐by‐sequencing may make SNP discovery and typing more cost‐effective (Davey et al., 2011; Morin et al., 2004).

Determining fine‐scale population structure is especially difficult for wide‐ranging species that inhabit remote areas where geographic boundaries are lacking or indistinct and events such as mating are difficult to observe. Such challenges were evident in studies of highly mobile carnivores with low densities and vast distributions (e.g., Cegelski, Waits, & Anderson, 2003; Kyle & Strobeck, 2001; Roy, Geffen, Smith, Ostrander, & Wayne, 1994; Sinclair et al., 2001). However, genetic approaches have the potential to provide insight into problems in conservation (Allendorf et al., 2010). For example, SNPs may be well suited to the study of fine‐scale structure (Haasl & Payseur, 2011; Liu, Chen, Wang, Oh, & Zhao, 2005; Rengmark, Slettan, Skaala, Lie, & Lingaas, 2006; Ryynanen, Tonteri, Vasemagi, & Primmer, 2007) because even a small number of SNPs can possess high information content (Lao, van Duijn, Kersbergen, de Knijff, & Kayser, 2006; Rosenberg, 2003). Thus, SNPs may improve the study and understanding of population genetic structure in such species.

Subpopulations of polar bears (*Ursus maritimus*) were first described using seasonal site fidelity of individuals to geographic areas. The basis for these subpopulations was inferred by: (i) mark–recapture movement studies, which compared the distance between capture and recapture locations of bears during the onshore period (Stirling, Lunn, Iacozza, Elliott, & Obbard, 2004; Taylor & Lee, 1995), (ii) the return of marked animals in subsistence harvest through a comparison of the displacement between capture and harvest location, and (iii) female‐based radiotelemetry studies, which provides year‐round information on movement and site fidelity (Bethke, Taylor, Amstrup, & Messier, 1996; Mauritzen et al., 2002; Obbard & Middel, 2012; Taylor et al., 2001). In Hudson Bay, where polar bears reside at the southern extent of their distribution, this information has led to the designation of three separate subpopulations for management purposes: Foxe Basin (FB), Southern Hudson Bay (SH), and Western Hudson Bay (WH). The region is ice‐covered from late fall to early summer and ice‐free during the rest of the year. In the spring during the ice‐covered period, polar bears are actively seeking mates. When the ice melts, bears are forced ashore (Amstrup, Marcot, & Douglas, 2008; Sahanatien, Peacock, & Derocher, 2015; Stirling et al., 2004) and show a high degree of fidelity to terrestrial sites including denning areas (Derocher & Stirling, 1990; Peacock, Derocher, Lunn, & Obbard, 2010; Stirling et al., 2004).

Previous studies of polar bear population genetics found genetic differentiation at both the global scale (Malenfant, Davis, Cullingham & Coltman, 2016; Paetkau et al., 1999; Peacock et al., 2015) and regional scale (Campagna et al., 2013; Crompton, Obbard, Petersen, & Wilson, 2008, 2014). Global‐scale studies generally supported the currently designated 19 subpopulations used for management purposes (Obbard, Thiemann, Peacock, & DeBruyn, 2010; Paetkau et al., 1999; Peacock et al., 2015), whereas regional studies show differentiation between adjacent subpopulations (e.g., Campagna et al., 2013). Although the first global‐scale studies defined the Hudson Bay region as a single unique genetic cluster (Paetkau et al., 1999), this study did not include samples from SH, and more recent fine‐scale studies that included samples from SH (Crompton et al., 2008, 2014; Malenfant et al., 2016; Peacock et al., 2015) identified substructure within Hudson Bay, specifically, a unique population in James Bay.

These population genetic studies varied in the distribution and density of sampling (e.g., Paetkau et al., 1999; *n* = 473; Crompton et al., 2008, 2014; *n* = 377, 2014; Campagna et al., 2013; *n* = 361; Peacock et al., 2015; *n* = 2,748; Malenfant et al., 2016; *n* = 495) and used small panels of microsatellite markers and mitochondrial DNA sequence data (e.g., Campagna et al., 2013; Malenfant et al., 2016; Peacock et al., 2015). The few studies that used SNPs used a small number of markers and sampling of only two to four subpopulations (Cronin et al., 2014; Miller et al., 2012); with the exception of Malenfant, Coltman & Davis (2015), who used 5,441 SNP markers, but only 78 individuals from 13 of the 19 worldwide subpopulations, including only 18 individuals from the Hudson Bay region.

The objective of our study was to examine fine‐scale subpopulation genetic structure of polar bears in the Hudson Bay region using a large number of SNP markers and a large number of continuously distributed samples, and to compare findings to past genetic studies and to the current subpopulation designations.

## 2 Methods

### 2.1 Study area and sampling

We used blood and skin samples from FB, SH, and WH polar bears as well as from the adjacent Davis Strait subpopulation (DS) because of its intermediate genetic relationship to the Hudson Bay region and the Canadian Archipelago (Malenfant et al., 2016; Obbard et al., 2010; Paetkau et al., 1999; Peacock et al., 2015). Samples were collected between 2004 and 2010 for SH (*n* = 112) and WH (*n* = 120) from capture–recapture studies conducted by the Ontario Ministry of Natural Resources and Forestry and Environment and Climate Change Canada, respectively. FB (*n* = 119) and DS (*n* = 63) samples were provided by the Governments of Nunavut (Department of Environment) and Newfoundland and Labrador (Department of Environment and Conservation) from capture–recapture studies, defense of life and property kills, and subsistence harvest. Capture and handling protocols were consistent with the guidelines of the Canadian Council on Animal Care and approved annually by Animal Care Committees of the Ontario Ministry of Natural Resources and Forestry, Environment and Climate Change Canada, and Quebec Wildlife Department for Animal Care (Ministère des Forêts, de la Faune et des Parcs Direction de la biodiversité et des maladies de la faune).

### 2.2 DNA extraction and genotyping

DNA was extracted using DNeasy Blood and Tissue kits (Qiagen, Hilden, Germany) following the manufacturers recommended protocol. SNP genotypes were obtained using a custom‐designed (Malenfant, Coltman & Davis (2015)), 9K Illumina Infinium BeadChip (Illumina, San Diego, CA, USA) processed by Delta Genomics (Edmonton, Canada). SNP genotypes were called using GenomeStudio 2011.1 (Genotyping Module 1.9; Illumina). Individuals with call rates of <0.9 were removed. Loci on the SNP chip were derived from both transcriptome and RAD sequencing (Malenfant, Coltman & Davis (2015)). Our focus and interest was to detect patterns of variation at likely neutral markers so only RAD loci were used for this study. Of 3,411 RAD SNPs that were polymorphic on the chip, loci were retained that had good clustering, high (>0.9) call rates, and were not X‐linked. Additional loci were removed with low minor allele frequencies (<0.01), high rates of missing data (>0.05) and those that were in linkage disequilibrium (LD). LD was assessed using a window of 10 SNPs that slides over by 1 SNP to prune at a cutoff of *r* ^2^ = 0.5 as implemented in a custom version of PLINK 1.07 (Malenfant, Coltman & Davis (2015), Purcell et al., 2007). There were no known first‐degree relationships among individuals in WH (Malenfant, Coltman, Richardson, et al. (2015)), SH, or FB. Relationships were not known from field observations for DS individuals.

### 2.3 Population structure

To investigate the pattern of population structure, we used discriminant analysis of principal components (DAPC, Jombart, Devillard, & Balloux, 2010) and STRUCTURE (Pritchard, Stephens, & Donnelly, 2000).

#### 2.3.1 Discriminant analysis of principal components

To identify and describe clusters of genetically similar individuals, we conducted a DAPC using the package adegenet (version 2.0‐0) (Jombart & Ahmed, 2011) implemented in the statistical program R version 3.2.4 (R Development Core Team, 2014). This approach transforms multilocus genotype data using principal component analysis (PCA) to derive the uncorrelated variables that serve as input for discriminant analysis (DA). In the assessment of population structure, the DA aims to maximize among‐group variation and minimize within‐group variation. In contrast to Bayesian clustering methods, DAPC does not require a population genetic model (Hardy–Weinberg or gametic equilibrium), nor is it as computationally intense and it is better at handling hierarchical structure or clinal variation caused by isolation by distance (Jombart et al., 2010; Kalinowski, 2011). We compared the results of DAPCs performed with and without prior groups assigned based on capture location (all individuals sampled within a specific subpopulation) (e.g., Möst et al., 2015; Pometti, Bessega, Saidman, & Vilardi, 2014; Quéméré et al., 2016). For DAPCs without prior information, the function *find.clusters()* in adegenet was used to determine the optimal number of clusters in our dataset. Specifically, we ran successive *k*‐means clustering with increasing number of clusters (*K* = 1–15 clusters) and used the *diffNgroup* option to identify the sharp changes in fit of models (measured using the Bayesian information criterion (BIC)) with different number of clusters (e.g., Buchalski et al., 2016; Vallejo‐Marin & Lye, 2013). We conducted 20 iterations to assess the stability in detection of the number of clusters. To prevent overfitting when conducting the DAPC, the number of principal components to retain was determined with a cross‐validation approach as implemented by the function *xvalDapc()* (e.g., Campoy et al., 2016; Van Cann, Virgilio, Jordaens, & De Meyer, 2015; Virgilio et al., 2015) with 100 repetitions.

#### 2.3.2 STRUCTURE analysis and postprocessing

Using STRUCTURE (Pritchard et al., 2000), we performed five independent runs for *K* = 1–10. We employed an admixture model with correlated allele frequencies using no location prior. Runs were performed for 150,000 Markov Chain Monte Carlo (MCMC) repetitions (including 50,000 burn‐in iterations). STRUCTURE output was analyzed using STRUCTURE HARVESTER version 0.693 (Earl & Vonholdt, 2012), and membership plots were visualized using CLUMPP 1.1.2 (Jakobsson & Rosenberg, 2007) and DISTRUCT 1.1 (Rosenberg, 2003).

Using the LnP(*K*) plots from STRUCTURE HARVESTER, optimal number of genetic clusters (*K*) was determined as the smallest value of *K* that captured the major structure of the dataset while maintaining small differences in likelihoods (i.e., plateau method; Pritchard et al., 2000). We opted for the plateau criterion because the ΔK method (Evanno et al. 2005) can be biased toward detection of the first structural level in the data, and miss fine‐scale structure (Gao, Bryc, & Bustamante, 2011; Goedbloed et al., 2013; Waples & Gaggiotti, 2006; Welch et al., 2012).

For our analyses, an ancestry (Q) cutoff was applied to identify strongly assigned and admixed individuals. Based on the five independent runs of STRUCTURE, individuals were identified as strongly assigned to a cluster if their mean Q was equal to or greater than 0.80. Sampling locations of strongly assigned individuals were plotted to visually assess similarity in geographic sampling location and genetic cluster assignment, and to examine how assignment relates to the site fidelity associated with capture location. These geographic plots were created with ArcMap version 10.1 (ESRI, Redlands, CA, USA), and pie graphs depicting the proportion of individuals sampled in each subpopulation that were strongly assigned/unassigned to each of *K* clusters were generated using Microsoft Excel 2011 (Microsoft Corporation, Redmond, WA, USA).

We also examined population structure using the spatial method of TESS3 version 1.0 (Caye, Deist, Martins, Michel, & François, 2016). We employed the associated TESS3r R package to perform five independent randomly seeded runs for *K* = 1–10 (tolerance = 1 × 10^−7^, max. iterations = 1,000). Using the cross‐entropy criterion with 5% of genotypes masked, the optimal *K*‐value was selected based on identification of the single run with the lowest cross‐entropy across 50 total runs.

### 2.4 Analysis of genetic variation

Observed heterozygosity (H_OBS_), expected heterozygosity (H_EXP_), inbreeding coefficients (*F*_IS_), and *F*_ST_ were calculated for capture location (all individuals sampled within a specific subpopulation) using GenoDive 2.0b27 (Meirmans & Van Tienderen, 2004). To test for significant F_IS_ values and departures from Hardy–Weinberg equilibrium, we used least squares and 1,000 permutations. A level of significance of α = 0.05 was used for all tests, with a Holm correction (Holm, 1979) for multiple tests where appropriate. AMOVA‐based pairwise *F*_ST_ values and *p*‐values were calculated using GenoDive's genetic differentiation option with default settings and 1,000 permutations. We estimated allelic richness and private allelic richness for each genetic cluster using rarefaction in ADZE 1.0 (Szpiech, Jakobsson, & Rosenberg, 2008) using a missing‐data cutoff of 0.05.

## 3 Results

The final dataset consisted of 2,603 loci typed in 414 individual polar bears (210 males and 204 females) sampled across the four current subpopulations (WH, SH, FB, and DS).

**Figure 1:**
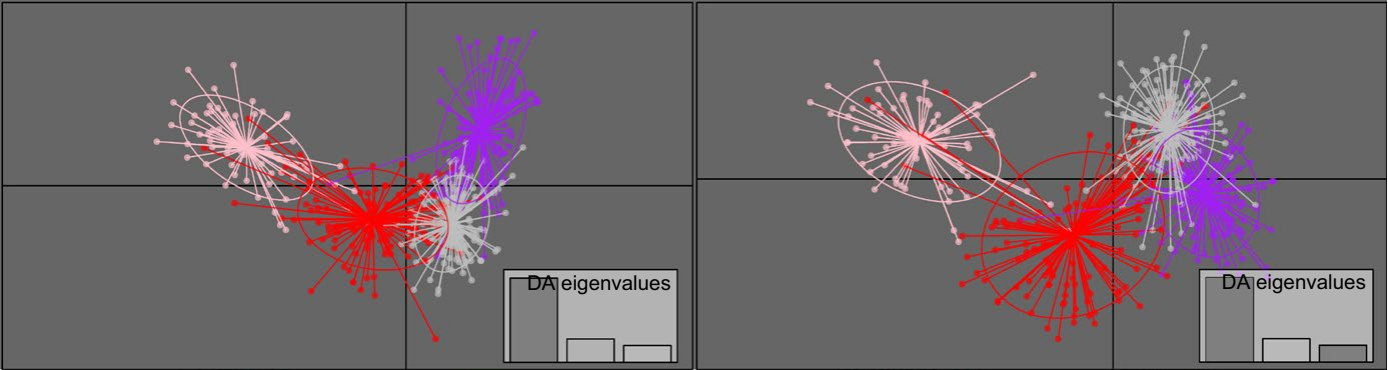
Scatterplots from discriminant analysis of principal components (DAPC) predefined by capture location of individuals in designated subpopulations of polar bears (*n* = 414) in the Hudson Bay region. The graph represents individuals as dots with the majority within inertia ellipses. Eigenvalues of the analysis are displayed in the inset. Subpopulations are labeled by different colors and abbreviated for the following, FB; Foxe Basin (in red), DS; Davis Strait (in pink), SH; Southern Hudson Bay (in purple), WH; Western Hudson Bay (in gray). The discriminant functions are one (*x*‐axis) and two (*y*‐axis) (left) and one (*x*‐axis) and three (*y*‐axis) (right)

**Figure 2:**
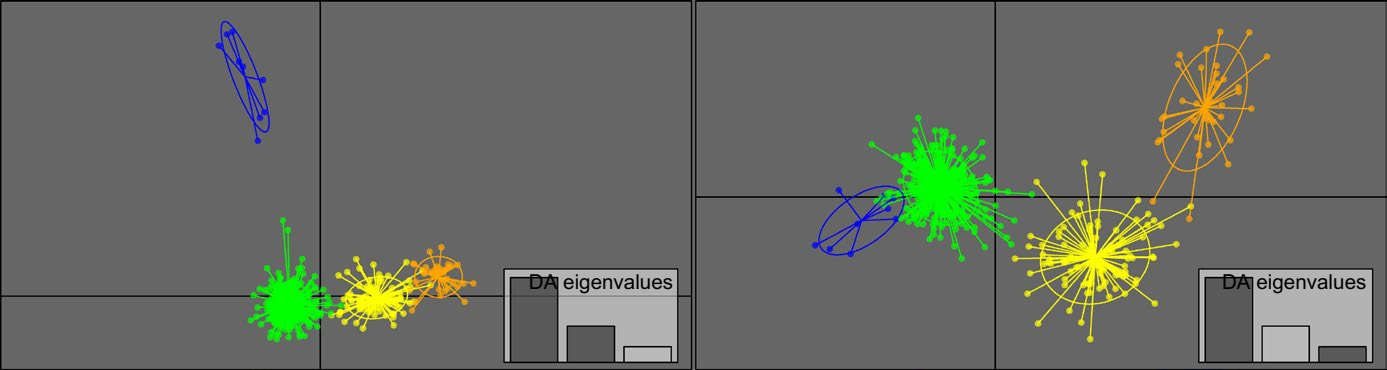
Scatterplots from discriminant analysis of principal components (DAPC) based on independent analysis of polar bears (*n* = 414) in the Hudson Bay region. The graph represents individuals as dots with the majority within inertia ellipses. Eigenvalues of the analysis are displayed in the inset. The discriminant functions are one (*x*‐axis) and two (*y*‐axis) (left), and one (*x*‐axis) and three (*y*‐axis) (right). Identified groups are color‐coded as Western (in green), Northern (in yellow), Southeast (in blue), Northeast (in orange)

The DAPC using capture location to define groups demonstrated substantial overlap between clusters (Figure 1). Without predefined groups and using traditional assessment for the optimal *K*, the BIC plot displayed the lowest value at *K* = 2. However, the *diffNgroups* option supported the presence of four genetic clusters in 16 of 20 runs. At *K* = 4, the first two discriminant functions clearly separated one cluster from the remaining groups (Figure 2, left), while the third discriminant function differentiated among the remaining three clusters (Figure 2, right). DAPC scatterplots for *K* = 2–6 are shown in Figure S2.

**Figure 3:**
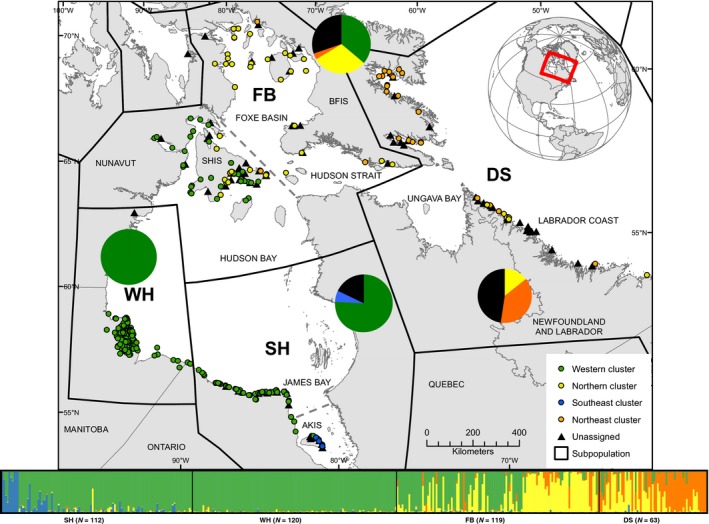
Population structure of polar bears in the Hudson Bay region derived from 414 samples using 2,603 SNPs at *K* = 4 depicted as geographic (top) and admixture (below) plots. The geographic plot illustrates the capture location of individuals and their assignment (indicated by color) to one of four clusters (Western, Northern, Southeast, Northeast); unassigned individuals are shown in black. The admixture plot shows each individual by a thin vertical line, which is divided into *K* colored segments indicating an individual's estimated membership in *K* clusters. Black lines indicate current subpopulation boundaries (WH, Western Hudson Bay; SH, Southern Hudson Bay; FB, Foxe Basin; DS, Davis Strait). Dashed gray line indicates proposed change in boundary lines. Subpopulation names and sample sizes are labeled on the admixture plot. Overlaid pie charts show the proportion of individuals strongly assigned (indicated by color) or unassigned (indicated by color black) to each subpopulation designation. Regional islands have been abbreviated and include AKIS, Akimiski Island; SHIS, Southampton Island; and BFIS, Baffin Island

Using STRUCTURE, we observed that the mean likelihood curve had nearly equal, yet increasing, likelihoods from *K* = 2 to *K* = 6 (Figure S3). However, *K* = 4 represented the smallest value of *K* that captured the majority of the structure in the dataset while maintaining small differences in likelihoods (Pritchard et al., 2000) and was chosen for further analyses (plots of additional *K* values are presented in the Figures S4–S8). Examination of the replicated runs of *K* = 4 showed two nearly equally likely solutions. However, only one solution was well supported when strongly assigned individuals were mapped by capture location (Figure 3) and showed concurrent assignment when compared to DAPC memberships at *K* = 4. The “alternate” solution was characterized by many individuals displaying large amounts of admixture (Figure S6).

We named the four genetic clusters identified by DAPC and STRUCTURE as: (i) Western—including individuals sampled in WH, SH (excluding Akimiski Island in James Bay), and southern FB (south of Southampton Island); (ii) Northern—individuals sampled in northern FB (Baffin Island) and DS (Labrador coast); (iii) Southeast—individuals from SH (Akimiski Island in James Bay); and iv) Northeast—individuals from DS (Baffin Island).

The cross‐entropy criterion implemented in TESS3 does not differentiate between DAPC identified Northern and Northeastern clusters. However, the TESS analysis did support a distinction between north of Southampton Island to the east and DS. Findings indicate *K*‐value of three (Figures S9 and S10), which corresponds to the identified Western, Northern, and Southeast genetic clusters. Samples from the Akimiski Island of James Bay region showed isolation from the other two genetic clusters (Figure S9).

Observed heterozygosity and expected heterozygosity values were low across capture location (Table 1). Observed heterozygosity, expected heterozygosity, and *F*_IS_ estimates and tests were not performed for genetic clusters as the removal of admixed individuals results in an unequal sample size for comparison to values calculated using capture locations. In addition, STRUCTURE derived clusters that include strongly assigned individuals will by definition have lower inbreeding coefficients. We found more inbreeding than expected in SH, FB, and DS and the opposite to be true in WH. Each showed significant departures from Hardy–Weinberg equilibrium (Table 1).

**Table 1:** Observed heterozygosity (*H*_OBS_), expected heterozygosity (*H*_EXP_), and inbreeding coefficient (*F*_IS_) estimates by capture location in the Hudson Bay region

Capture location (*n* = 414)	*n*	*H*_OBS_	*H*_EXP_	*F*_IS_
WH	120	0.245	0.244	−0.005*
SH	112	0.243	0.244	0.005*
FB	119	0.244	0.247	0.012*
DS	63	0.250	0.252	0.009*

WH, Western Hudson Bay; SH, Southern Hudson Bay; FB, Foxe Basin; DS, Davis Strait.

Sample sizes are indicated. Significance at alpha of 0.05 after Holm correction is indicated by *.

Pairwise values of *F*_ST_ by capture location ranged from 0.007 to 0.02 (Table 2). The highest values were between DS/SH and DS/WH and only slightly greater than DS/FB (Table 2). Lower amounts of differentiation were found between SH/WH, SH/FB, and WH/FB. *F*_ST_ values based on genetic clusters exceed those between capture locations by approximately one order of magnitude, ranging between 0.014 and 0.101 (Table 2). The greatest population differentiation was between the Southeast to Northern and Northeast clusters.

**Table 2:** Comparison of pairwise *F*_ST_ values at the capture location and genetic cluster level for polar bears in the Hudson Bay region of Canada

Capture location	WH‐SH	WH‐FB	WH‐DS	SH‐FB	SH‐DS	FB‐DS
	0.004	0.006	0.020	0.007	0.020	0.010

Genetic cluster	W‐N	W‐SE	W‐NE	N‐SE	N‐NE	SE‐NE
	0.014	0.074	0.036	0.083	0.024	0.101

WH, Western Hudson Bay; SH, Southern Hudson Bay; FB, Foxe Basin; DS, Davis Strait; W, Western; N, Northern; SE, Southeast; NE, Northeast.

Sample size is indicated by capture location (total = 414; WH = 120, SH = 112, FB = 119, DS = 63) and genetic cluster based on strong assignment at *K* = 4 (total = 328; W = 248, N = 45, SE = 7, NE = 28). All F_ST_ values were significant after Holm correction (Holm‐corrected *p* ≤ .006).

Measures for genetic diversity were standardized relative to our smallest clusters (i.e., Southeast cluster *n* = 14 chromosomes; seven diploid individuals). Allelic richness was high in each of the genetic clusters, whereas the private allelic richness was low across genetic clusters (Table 3).

**Table 3:** Allelic richness and private allelic richness mean and standard error (*SE*) are presented at standardized sample size of 14 chromosomes for each genetic cluster of polar bears in the Hudson Bay region of Canada

	Western		Northern		Southeast		Northeast	
	Mean	*SE*	Mean	*SE*	Mean	*SE*	Mean	*SE*
Allelic richness	1.73	0.0949	1.73	0.100	1.58	0.244	1.75	0.097
Private allelic richness	0.034	0.007	0.033	0.006	0.015	0.005	0.059	0.019

In total, 2,601 loci were used. The following genetic clusters are based on strong assignment at *K* = 4 (total = 328; Western = 248, Northern = 45, Southeast = 7, Northeast = 28).

## 4 Discussion

Using powerful tools, such as genetic markers, to aid in clarifying relationships between individuals can add to our understanding and inform planning and future management actions. In this study, we found that SNPs identified fine‐scale structure in a set of thoroughly sampled individuals in the Hudson Bay region. Three independent methods identified genetic clusters within the Hudson Bay region that differ from both current management boundaries, and from previous studies of range‐wide and fine‐scale genetic structure in Hudson Bay (Crompton et al., 2008, 2014; Malenfant et al., 2016; Paetkau et al., 1999; Peacock et al., 2015).

### 4.1 Molecular data support designation of four subpopulations

The consensus across methods suggests *K* = 4 best describes the region's population structure. This conclusion is based on the combined lines of evidence from DAPC (using a method that is most suitable for data with hierarchical structure), STRUCTURE, and geographic concordance of individual genetic assignments in relation to capture locations (Figure 3).

With respect to the DAPC analysis, traditional assessment involving visual inspection of BIC values would suggest *K* = 2 (Figure S1). However, implementation of the *diffNgroup* option, which automates cluster identification by considering sudden rates of change in BIC values between a different number of clusters, found four groups. This method has been used previously to define groups in extant *Mimulus* species (Vallejo‐Marin & Lye, 2013). Similarly, simple implementation of the ΔK method (Evanno et al. 2005) to our STRUCTURE data would suggest *K* = 2. However, the efficacy of the ΔK method for detecting hierarchical population structure is debatable (Gao et al., 2011; Goedbloed et al., 2013; Waples & Gaggiotti, 2006; Welch et al., 2012). Evidence of hierarchical structure in our dataset is demonstrated by the increasing likelihood values of our STRUTURE runs (Figure S3); as well by the geographic concordance of individual genetic assignments in relation to capture locations (Figure 3). Although STRUCTURE produced two almost equal solutions derived at *K* = 4, the result presented in Figure 3 is more biologically plausible than the alternative case where admixture is present in more than half of the FB subpopulation (Figure S6). This first STRUCTURE result also stands in agreement with the clusters found by DAPC methods. STRUCTURE's difficulty in converging onto a single solution may have risen due to an underrepresentation of DS and/or lack of sampling outside of the Hudson Bay region. Both may have improved the program's ability to parse the ancestry of individuals.

In contrast to DAPC and STRUCTURE, TESS3 identified a maximum of three clusters in the Hudson Bay region (Figure S9). These groups correspond to the Western and Southeast clusters and then a single group combining the Northern and Northeast clusters. The analysis indicates spatially a division north of Southampton Island. It is important to note that TESS directly incorporates spatial information when determining the number of genetic clusters in a dataset. Therefore, given that the *F*_ST_ values for the Northern cluster tend to be lower (Table 2) and that individuals highly assigned to the Northern and Northeast clusters are dispersed along the Labrador coast and within Foxe Basin (Figure 3), it is possible that the model was not able to differentiate between these two groups in a spatially explicit manner. In a similar case, Fenderson, Kovach, Litvaitis, and Litvaitis (2011) hypothesized TESS2's lack of detection being linked to an interaction parameter between spatial coordinates and genetic data used in the algorithm, TESS3 may be affected in the same way. At this level of analysis, it is understandable that different methods would have varying sensitivities to detecting structure.

### 4.2 Comparison to previous studies

Our conclusions regarding the number of genetic clusters and their geographic locations differ from previous studies. These differences may be attributed to the larger number of markers, greater sample sizes, and more even distribution of sampling across the landscape used in our study. In particular, our sampling was continuous and evenly distributed within SH, a subpopulation that was not included in Paetkau et al. (1999) and was also more extensive than in either Crompton et al. (2008, 2014), or Paetkau et al. (1999), Peacock et al. (2015), or Malenfant et al. (2016) with respect to northern FB.

However, although different from previous studies our results are in line with the structure they highlighted. The initial research by Paetkau et al. (1999) suggested population structure in the Hudson Bay region and the likely presence of two genetic clusters. Although Paetkau et al. (1999) identified a single genetic cluster in Hudson Bay that included DS; DS was recognized as an intermediate subpopulation, which is hypothesized to connect the Canadian Archipelago to Hudson Bay. The population structure in our analysis seen at *K* = 2 (Figures S2 and S4) and spatially by TESS3 (Figure S9) demonstrates this first degree of differentiation, grouping WH, SH, and southern FB in one cluster and northern FB, and DS in a second. Thus, suggesting the Canadian Archipelago ancestry is the strongest signal within the region.

Previous evidence of fine‐scale structure in the Hudson Bay region was found by Crompton et al. (2008, 2014), Peacock et al. (2015), and Malenfant et al. (2016). These authors identified an additional cluster within SH in James Bay. Our results are consistent with theirs; even with only a small number of individuals sampled in James Bay, our methodologies consistently detected structure at the southern extent of our sampling. However, in addition to a Southeast cluster, we found a novel genetic cluster in the northern areas of FB. The presence of this fourth genetic cluster is further corroborated by an extensive study of telemetry in polar bears exclusively from FB. Sahanatien et al. (2015) detected population structure in the form of distinct movement patterns, demonstrating that bears of FB segregate into three patterns. One of these behavioral patterns corresponds to the genetic distinctiveness we detected in the northern area of FB (Figures 2 and 3). A division within FB is further suggested by a study examining the return of Inuit marked harvested bears (Stirling & Ramsay, 1986), indicating a split in the north and south. Collectively these lines of evidence support our study's justification of *K* = 4 in the Hudson Bay region.

### 4.3 Origins of population structure

The presence of genetic structure in polar bears could be a result of geographic features such as polynyas or landmasses that can act as barriers to gene flow (Paetkau et al., 1999) or could be linked to characteristics of sea‐ice habitat, which can influence polar bear movement (Derocher & Stirling, 1990; Stirling et al., 2004). For example, the split within FB that we found may be due to the pattern of sea‐ice breakup in Foxe Basin as the northern portion of the basin retains ice longer (Stewart & Barber, 2010) and the physical presence of Southampton Island may separate the bears for part of the year. Telemetry data of polar bears collared in FB indicate three space‐use patterns with individuals preferentially inhabiting either Hudson Bay, Foxe Basin, or Hudson Strait (Sahanatien et al., 2015). Similar to many other species, natal philopatry may also be a driving factor in the development of genetic structure (e.g., snow goose, Avise, Alisauskas, Nelson, & Ankney, 1992; prairie voles, McGuire, Getz, Hofmann, Pizzuto, & Frase, 1993; walleye, Stepien & Faber, 1998; Antarctic seals, Davis, Stirling, Strobeck, & Coltman, 2008). Although knowledge on the subject is not extensive, polar bears do exhibit seasonal site fidelity throughout their range (Born, Wiig, & Thomassen, 1997; Cherry, Derocher, Thiemann, & Lunn, 2013; Derocher & Stirling, 1990; Harrington, 1968; Lone, Aars, & Ims, 2013; Ramsay & Stirling, 1990). This could be the case for individuals with membership in the Southeast cluster, which were exclusively sampled in James Bay (Akimiski Island). Satellite telemetry data indicate that few animals enter or exit James Bay (Obbard & Middel, 2012), suggesting that the observed genetic differentiation is likely a product of behaviors such as site fidelity during the mating season.

Regardless of the cause, evidence for fine‐scale structure suggests that polar bears in the Hudson Bay region are not panmictic. Mating among polar bears of the region is non‐random, which has given rise to the observed genetic structure. Gene flow occurs when dispersal is effective (i.e., results in genetic exchange) (Slatkin, 1987), and can occur with some randomness to link subpopulations (Waples & Gaggiotti, 2006). Paetkau et al. (1999) reported that global‐scale gene flow was uneven between polar bear subpopulations, and similarly our heterozygosity and *F*_ST_ values suggest varying levels of gene flow occurs between clusters within the Hudson Bay region.

Based on pairwise *F*_ST_ estimates (Table 2), genetic relationships are closest between bears of the Northern, Western, and Northeast clusters. However, an area of isolation among bears is evident in the James Bay region (specifically Akimiski Island) as the strongest differentiation was between the Southeast cluster and the other three clusters (Table 2). It is possible a group of bears in this region has become demographically and geographically limited, similar to what is observed in Norwegian Bay (Paetkau et al., 1999), and is reflected in the limited genetic diversity (Table 1).

### 4.4 Comparison between subpopulation designation and genetic clusters

Although the current subpopulation designations were developed without genetic data, they were assumed to capture population structure. However, we find that the capture location of individuals assigned to the four genetic clusters is not reflected by the current subpopulation boundaries (Figure 3). These results are echoed by examination of DAPC plots using predefined groups (Figure 1), where we see substantial overlap between clusters defined by capture location. Such grouping would suggest weak genetic structuring overall. Quantitatively, *F*_ST_ estimates based on capture location demonstrate that there is little genetic differentiation between subpopulations, which is in stark contrast to comparisons of these values to those calculated using the derived genetic clusters. The current subpopulation designation boundaries used for management purposes are not reflective of genetic structure.

### 4.5 Management

At the global scale, large carnivores are increasingly becoming endangered (Cardillo et al., 2005; Gittlemen & Gompper, 2001); however, efforts to identify and address declines are hampered by limited data (Kendall et al., 2009). SNPs are a powerful resource to aid in the challenge of establishing clear subpopulation boundaries for management of wide‐ranging species. Traditionally, an a *priori* approach using geographic and political information is often implemented (Cegelski et al., 2003; Nagy et al., 2011; Vähä, Erkinaro, Niemelä, & Primmer, 2007). However, genetic structure detected in analyses using geopolitically informed boundaries could mask true genetic structure and mislead interpretations of genetic clusters (Meirmans, 2015). For polar bears, subpopulations were designed for harvest purposes, influenced by jurisdictional boundaries and further developed based on a diversity of inputs that evolved with advances in technology to include telemetry data (Bethke et al., 1996; Obbard & Middel, 2012; Taylor & Lee, 1995; Taylor et al., 2001). The subpopulations have been effective in their primary function for harvest management.

Our SNP‐based analyses indicate a discrepancy between capture location and genetic cluster categories. Genetic data could be used to further delineate subpopulations, and if so, we would recommend adjusting the boundary lines to include a division north of Southampton Island, and below the mouth of James Bay (Figure 3), which are similarly seen in Figure S9. From a genetic and ecological perspective, these would reflect the genetic discontinuity within FB and SH and complement telemetry data from Obbard and Middel (2012) and Sahanatien et al. (2015), which together suggest support of a relationship between genetic differentiation and space‐use. As polar bear management initiatives shift toward conservation efforts, ecological and genetic perspectives that recognize behaviors and spatial organization on the sea ice during an important time of year when feeding and mating occur may be valuable for future assessments.

### 4.6 Conclusions and future directions

Fine‐scale structure was evident in polar bears in the Hudson Bay region, which differs from the subpopulation management designations currently used. We suggest SNPs will be useful when subpopulation definitions and delineation methods include genetic differentiation. We find four clusters—Western, Southeast, Northern, and Northeast—that best describe the current population structure of polar bears in the Hudson Bay region. With projected sea‐ice changes, polar bear management issues need to transition from being harvest focused to conservation based to better identify important habitats to preserve genetic variation.

## Data accessibility

SNP data are available on Dryad (doi: 10.5061/dryad.1719f)
