# Supplementary material for: Assessing polar bear (Ursus maritimus) population structure in the Hudson Bay region using SNPs
Source: Ecol Evol. 2016 Oct 28;6(23):8474–84. doi: 10.1002/ece3.2563 (PMC5167041; doi:10.1002/ece3.2563)
Supplement: Supplementary file 1 [file ECE3-6-8474-s001.docx]

**Supporting Information**

**Supporting Information Figure 1** Bayesian information criterion (BIC) values plotted for number of clusters ranging from K = 1 to 40 derived from discriminant analysis of principal components (DAPC).

**Supporting Information Figure 2** Scatterplots from discriminant analysis of principal components (DAPC) from K= 2 to K = 6 for polar bears (n = 414) in the Hudson Bay region. The plots are arranged by ascending K, starting at the top left with K = 2 and the top right with K = 3. Discriminant functions one (x-axis) and two (y-axis) are presented on the left and discriminant functions one (x-axis) and three (y-axis) are on the right where applicable. Eigenvalues of the analysis are displayed in inset. Within each plot, an individual is represented as a dot with the majority of dots within inertia ellipses. Genetic clusters are labeled by different colours (see legend).

**Supporting Information Figure 3** Mean LnP(K) and Delta K plots for SNP-based (n = 2603) STRUCTURE analysis (n = 414) averaged over five repetitions at K = 1 to 10.

**Supporting Information Figure 4** Population structure of polar bears in the Hudson Bay region derived from 414 samples using 2603 SNPs at K = 2 depicted as geographical (top) and admixture (below) plots. The geographical plot illustrates the capture location of individuals and their assignment (indicated by colour) to one of two clusters (Western, and Northern); unassigned individuals are shown in black. The admixture plot shows each individual by a thin vertical line, which is divided into K coloured segments indicating an individual’s estimated membership in K clusters. Black lines indicate current subpopulation boundaries (WH, Western Hudson Bay; SH, Southern Hudson Bay; FB, Foxe Basin; DS, Davis Strait). Subpopulation names and sample sizes are labelled on the admixture plot. Overlaid pie charts show the proportion of individuals strongly assigned (indicated by colour) or unassigned (indicated by colour black) to each subpopulation designation. Regional islands have been abbreviated and include AKIS, Akimiski Island; SHIS, Southampton Island; and BFIS, Baffin Island.

**Supporting Information Figure 5** Population structure of polar bears in the Hudson Bay region derived from 414 samples using 2603 SNPs at K = 3 depicted as geographical (top) and admixture (below) plots. The geographical plot illustrates the capture location of individuals and their assignment (indicated by colour) to one of three clusters (Western, Northern, Southeast); unassigned individuals are shown in black. The admixture plot shows each individual by a thin vertical line, which is divided into K coloured segments indicating an individual’s estimated membership in K clusters. Black lines indicate current subpopulation boundaries (WH, Western Hudson Bay; SH, Southern Hudson Bay; FB, Foxe Basin; DS, Davis Strait). Subpopulation names and sample sizes are labelled on the admixture plot. Overlaid pie charts show the proportion of individuals strongly assigned (indicated by colour) or unassigned (indicated by colour black) to each subpopulation designation. Regional islands have been abbreviated and include AKIS, Akimiski Island; SHIS, Southampton Island; and BFIS, Baffin Island.

**Supporting Information Figure 6** The alternate solution for population structure of polar bears in the Hudson Bay region derived from 414 samples using 2603 SNPs at K = 4 depicted as geographical (top) and admixture (below) plots. The geographical plot illustrates the capture location of individuals and their assignment (indicated by colour) to one of four clusters (Western, Northern, Southeast, Northeast); unassigned individuals are shown in black. The admixture plot shows each individual by a thin vertical line, which is divided into K coloured segments indicating an individual’s estimated membership in K clusters. Black lines indicate current subpopulation boundaries (WH, Western Hudson Bay; SH, Southern Hudson Bay; FB, Foxe Basin; DS, Davis Strait). Subpopulation names and sample sizes are labelled on the admixture plot. Overlaid pie charts show the proportion of individuals strongly assigned (indicated by colour) or unassigned (indicated by colour black) to each subpopulation designation. Regional islands have been abbreviated and include AKIS, Akimiski Island; SHIS, Southampton Island; and BFIS, Baffin Island.

**Supporting Information Figure 7** Population structure of polar bears in the Hudson Bay region derived from 414 samples using 2603 SNPs at K = 5 depicted as geographical (top) and admixture (below) plots. The geographical plot illustrates the capture location of individuals and their assignment (indicated by colour) to one of five clusters (Western, Northern, Southeast, Northeast, Central); unassigned individuals are shown in black. The admixture plot shows each individual by a thin vertical line, which is divided into K coloured segments indicating an individual’s estimated membership in K clusters. Black lines indicate current subpopulation boundaries (WH, Western Hudson Bay; SH, Southern Hudson Bay; FB, Foxe Basin; DS, Davis Strait). Subpopulation names and sample sizes are labelled on the admixture plot. Overlaid pie charts show the proportion of individuals strongly assigned (indicated by colour) or unassigned (indicated by colour black) to each subpopulation designation. Regional islands have been abbreviated and include AKIS, Akimiski Island; SHIS, Southampton Island; and BFIS, Baffin Island.

**Supporting Information Figure 8** Population structure of polar bears in the Hudson Bay region derived from 414 samples using 2603 SNPs at K = 6 depicted as geographical (top) and admixture (below) plots. The geographical plot illustrates the capture location of individuals and their assignment (indicated by colour) to one of six clusters (Western, Northern, Southeast, Northeast, Central, Southern); unassigned individuals are shown in black. The admixture plot shows each individual by a thin vertical line, which is divided into K coloured segments indicating an individual’s estimated membership in K clusters. Black lines indicate current subpopulation boundaries (WH, Western Hudson Bay; SH, Southern Hudson Bay; FB, Foxe Basin; DS, Davis Strait). Subpopulation names and sample sizes are labelled on the admixture plot. Overlaid pie charts show the proportion of individuals strongly assigned (indicated by colour) or unassigned (indicated by colour black) to each subpopulation designation. Regional islands have been abbreviated and include AKIS, Akimiski Island; SHIS, Southampton Island; and BFIS, Baffin Island.


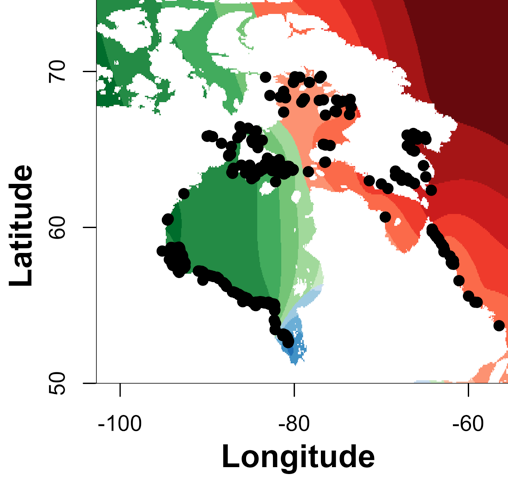


**Supporting Information Figure 9** Map of Canadian Arctic and Greenland highlighting results from TESS3 analysis of population structure of polar bears (n = 414) from the Hudson Bay region of Canada. Three genetic clusters are identified as the best K as seen in green, blue and red and individual samples are represented by black dots.


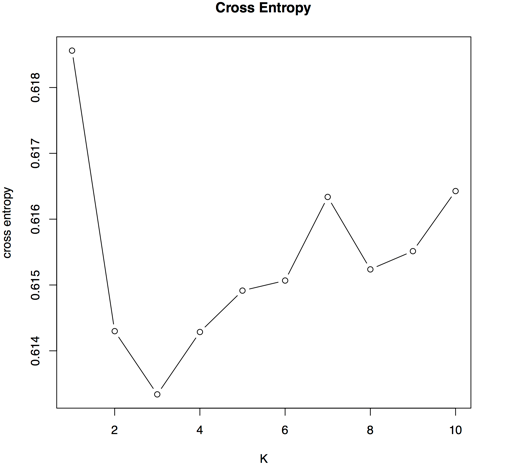


**Supporting Information Figure 10** Cross-entropy by K-value derived from TESS3 analysis of polar bears (n = 414) from the Hudson Bay region of Canada.
